# Basement membrane proteins in various arterial beds from individuals with and without type 2 diabetes mellitus: a proteome study

**DOI:** 10.1186/s12933-021-01375-7

**Published:** 2021-09-08

**Authors:** Lasse Bach Steffensen, Xenia Emilie Sinding Iversen, Rasmus Søgaard Hansen, Pia Søndergaard Jensen, Anne-Sofie Faarvang Thorsen, Jes Sanddal Lindholt, Lars Peter Schødt Riber, Hans Christian Beck, Lars Melholt Rasmussen

**Affiliations:** 1 Department of Clinical Biochemistry and Pharmacology, Odense University Hospital, Sdr. Boulevard 29, 5000 Odense, Denmark; 2 Centre for Individualized Medicine in Arterial Diseases (CIMA), Odense University Hospital, Odense, Denmark; 3 Unit of Cardiovascular and Renal Research, Department of Molecular Medicine, University of Southern Denmark, Odense, Denmark; 4 Department of Cardiac, Thoracic, and Vascular Surgery, Odense University Hospital, Odense, Denmark

**Keywords:** Proteomics, Mass spectrometry, Type 2 diabetes mellitus, Basement membrane, Artery

## Abstract

**Background:**

Basement membrane (BM) accumulation is a hallmark of micro-vessel disease in diabetes mellitus (DM). We previously reported marked upregulation of BM components in internal thoracic arteries (ITAs) from type 2 DM (T2DM) patients by mass spectrometry. Here, we first sought to determine if BM accumulation is a common feature of different arteries in T2DM, and second, to identify other effects of T2DM on the arterial proteome.

**Methods:**

Human arterial samples collected during heart and vascular surgery from well-characterized patients and stored in the Odense Artery Biobank were analysed by liquid chromatography coupled with tandem mass spectrometry (LC–MS/MS). We included ascending thoracic aortas (ATA) (*n* = 10 (type 2 DM, T2DM) and *n* = 10 (non-DM)); laser capture micro-dissected plaque- and media compartments from carotid plaques (*n* = 10 (T2DM) and *n* = 9 (non-DM)); and media- and adventitia compartments from ITAs (*n* = 9 (T2DM) and *n* = 7 (non-DM)).

**Results:**

We first extended our previous finding of BM accumulation in arteries from T2DM patients, as 7 of 12 pre-defined BM proteins were significantly upregulated in bulk ATAs consisting of > 90% media. Although less pronounced, BM components tended to be upregulated in the media of ITAs from T2DM patients, but not in the neighbouring adventitia. Overall, we did not detect effects on BM proteins in carotid plaques or in the plaque-associated media. Instead, complement factors, an RNA-binding protein and fibrinogens appeared to be regulated in these tissues from T2DM patients.

**Conclusion:**

Our results suggest that accumulation of BM proteins is a general phenomenon in the medial layer of non-atherosclerotic arteries in patients with T2DM. Moreover, we identify additional T2DM-associated effects on the arterial proteome, which requires validation in future studies.

**Supplementary Information:**

The online version contains supplementary material available at 10.1186/s12933-021-01375-7.

## Introduction

It is estimated that more than 463 million people worldwide are living with diabetes mellitus (DM) [1], and the global prevalence of DM continues to increase [2]. Atherosclerotic cardiovascular disease is the most frequent cause of morbidity and death in individuals with DM [3]. On the other hand, DM patients display partial protection from development of aneurysms [4]. Moreover, the arterial wall in diabetes has been shown to harbor several generalized pathologies such as linear media calcifications [5, 6], increased stiffness [7], accumulation of non-enzymatically glycated substances [8] and dysfunctional remodeling of coronary arteries [9]. These observations seem to indicate that macrovascular disease in diabetes contain specific generalized elements, which may lead to the clinical cardiovascular conditions. Putative alterations in the arterial proteome in diabetes may demonstrate associations relevant to these generalized changes and insight into the arterial protein composition may potentially lead to mechanistic insight, new treatment targets and novel biomarkers.

Current proteomic technology offers the possibility to achieve quantitative information on a large set of proteins in numerous samples, but studies addressing the human arterial proteome are limited. Reports from Mayr et al*.* provided a sequential extraction protocol on human arteries to characterize the protein content [10], and found extracellular matrix changes in relatively few abdominal aortic aneurysm lesions compared to non-aneurysmatic tissue [11]. Studies including larger numbers of patient samples have provided insight into vascular protein changes in relation to smoking, arterial stiffness and aneurysm growth [12–14]. Importantly, these studies have demonstrated that proteomic insight can be obtained by liquid chromatography-mass spectrometry (LC–MS) with high technical precision.

Our group previously reported that the arterial proteome is altered in bulk non-atherosclerotic internal thoracic arteries (ITA) from type 2 DM (T2DM) patients [15]. By a hypothesis-free LC–MS/MS-based proteomics approach, we reported significant enrichment of basement membrane (BM) components in these arteries [15]. This observation implied that BM accumulation is not limited to micro-vessels (e.g. in kidney and skin) in DM [15], but a general feature of arteries [15]. Interestingly, the *COL4A1/A2* locus encoding the ⍺1- and ⍺2-chains of collagen type IV (which comprise the scaffold of the arterial BM) is a highly replicated genome-wide association study (GWAS) hit for coronary- and peripheral artery disease [16–18]. This supports a causal role of the BM in atherosclerosis, and prompts the question of whether BM enrichment of arteries in diabetes could have atherosclerosis-promoting effects.

In this study, we investigated whether arterial BM accumulation is a common feature of arteries in T2DM individuals, i.e. both in non-diseased muscular- and elastic arteries, and during atherosclerosis development. Moreover, we extended the study with explorative analyses to identify novel T2DM-regulated arterial proteins. By combining laser-capture microdissection (LCM) and LC–MS/MS, we profiled the proteome from intimal-, medial-, and adventitial layers separately to assess compartment-specific effects of DM. This has not previously been done on human arterial samples.

## Materials and methods

### Patient samples

Arterial tissues from 55 individuals were retrieved from the Odense Artery Bank (OAB) [19]. The OAB consists of patients aged ≥ 18 years, who have undergone elective cardiovascular surgery, including aortic/mitral valve replacement, coronary artery bypass grafting (CABG) or carotid endarterectomy at the Department of Cardiac, Thoracic and Vascular Surgery, Odense University Hospital [13]. All participants have given written consent based on oral and written information, and OAB is approved by the local ethics committees (S-20100044).

**Fig. 1:**
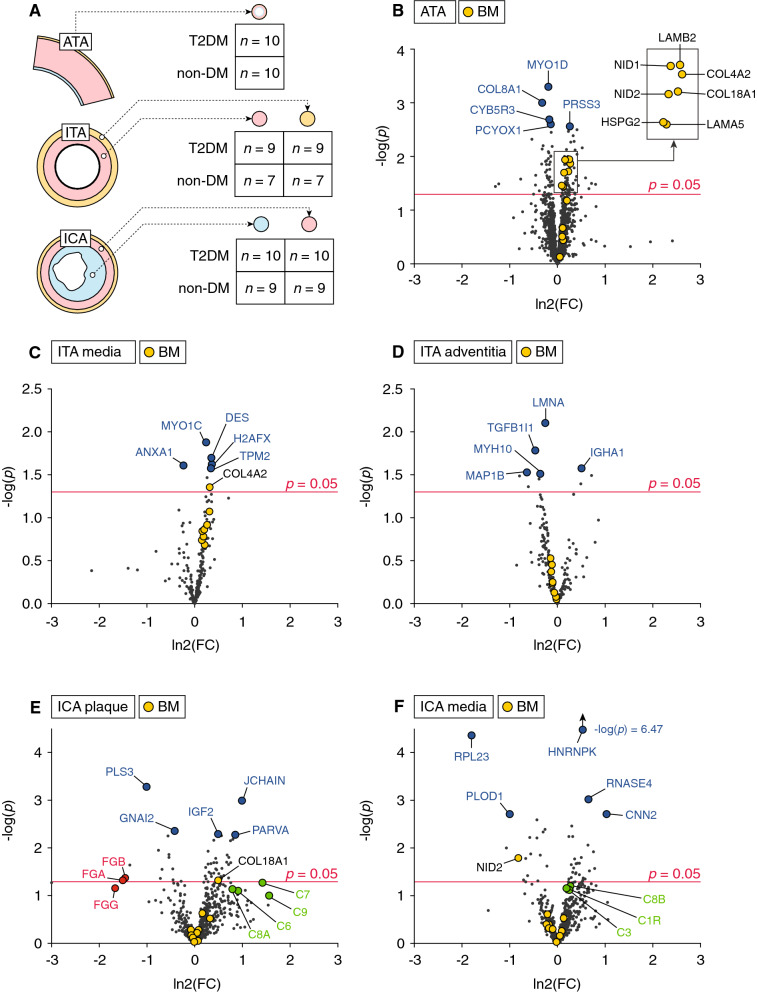
**A** Summary of samples analysed in the study. **B–F** Volcano plots showing regulation of all identified proteins in all tissues/compartments. The volcano plots graphically represent the effect of T2DM compared to non-DM on all proteins measured for each tissue type (each dot represents a unique protein). The plots show magnitude of change (fold change, FC) (x-axis) versus statistical significance (y-axis). FCs are ln2-transformed (i.e*.* upregulated proteins will have a ln2(FC) > 0 and vice versa), and the significance level is shown as the negative logarithm (base 10) of the *p* value (i.e*.* proteins with a value above ~ 1.3 have a *p* < 0.05). Pre-defined basement membrane (BM) proteins are shown as magnified yellow dots. The top 5 most significantly regulated proteins for each tissue/compartment are shown as magnified blue dots along with identities. In E, fibrinogens are shown as magnified red dots with identities. In **E**, **F** complement factors are shown as magnified green dots with identities. ATA: ascending thoracic aorta; ITA: internal thoracic artery; ICA: Internal carotid artery

In the present study, we analysed ascending thoracic aortas (ATA) from CABG (*n* = 10 (T2DM) and *n* = 10 (non-DM)); ITAs from CABG (*n* = 9 (T2DM) and *n* = 7 (non-DM)), and internal carotid arteries (ICAs) from carotid endarterectomies (*n* = 10 (T2DM) and *n* = 9 (non-DM)). Since medial- and adventitial layers of ITAs as well as plaque- and medial compartments from ICAs were analysed separately, we analysed a total of 90 samples (Fig. 1A).

### Tissue processing

Immediately after surgery the arterial tissue was formalin-fixed (~ 24 h in 4% buffered paraformaldehyde) before being transferred to a phosphate-buffered saline solution and subsequently embedded in paraffin (FFPE), sectioned (ITAs: 8 μm, ATAs: 10 μm and ICAs: 10 μm), and stored in the OAB (representative Weigert (elastin) and Masson trichrome stained sections are shown in Additional file 1: Figure S1).

Based on histological assessment, ITA sections had < 5% of the circumference covered with intimal lesions and ATAs sections consisted > 90% media and devoid of pathological intima thickening.

FFPE sections were deparaffinized in chloroform and serial sections were placed on membranes for LCM. Medial- and adventitial layers of ITA sections were collected in separate vials with adhesive lids by LCM performed with an MMI CellCut Laser (Molecular Machines and Industries GmbH, Munich, Germany) on an Olympus microscope (Olympus, Tokyo, Japan). Medial layer samples from ITAs were micro-dissected with some margin to the internal elastic lamina ensuring that samples were not contaminated by material from the intimal layer. Likewise, the plaque area and underlying medial layer of ICAs were separately collected. LCM samples were stored at − 20 °C.

### Preparation of samples for proteomic analysis

LCM-prepared ITA and ICA samples were prepared for proteomic analysis as described previously [20]. In brief, proteins were extracted using 4 mM triethyl ammonium bicarbonate (TEAB), 1 mM ethylenediamine tetraacetic acid (EDTA) and 0.002% zwittergent 3–16, incubated for 90 min a 98 °C followed by reduction and alkylation by sequential addition of dithiothreitol (DTT, 5 mM for 30 min at 50 °C) and iodoacetamide (IAA, 15 mM for 30 min at room temperature (RT) in the darkness). Proteins were isolated by acetone precipitation, solubilized in 20 µl 0.4 M TEAB and proteolytically processed by overnight incubation with trypsin (0.01 µg/µl) at 37 °C.

ATA samples were prepared for proteomic analysis also as previously described [21]. In brief, sections were deparaffinized in chloroform, and proteins were extracted using 1 M DTT, 0.2 M TEAB and 10% sodium dodecyl sulphate, and incubated at 99 °C for 20 min, and then at 80 °C for 120 min. Protein alkylation was performed by adding a 200 mM IAA solution to a final DTT/IAA concentration ratio of 1:3 and incubation at RT for 30 min in the darkness. The acetone-precipitated proteins were solubilized in 5 µL 8 M urea with 1 µg LysC and incubated at 30 °C for 4 h, followed by a further dilution to 1 M urea with 0.2 M TEAB, the addition of 2 µg trypsin, and an overnight incubation at 37 °C.

Tryptic peptides from ITA and ICA samples were isotopically labelled using 10-plex tandem mass tags (ThermoFisher Scientific, Waltham, Massachusetts, USA), and ATA samples were labelled using 11-plex tandem mass tags. For ICA samples, pools of all plaque samples (mass tag 131) and all media samples (mass tag 126) served as internal controls. For ITA samples, pools of all media samples (mass tag 126) and adventitia samples (mass tag 131) served as internal controls. For ATA samples, a pool of all samples (mass tag 126) served as internal control. Peptide samples from each of the three tissues (ICA, ITA and ATA) were randomly labelled with the remaining mass tags (127N, 127C, 128N, 128C, 129N, 129C, 130N, 130C and 131C). Tagged peptides were mixed into two mixed peptide samples for ATAs, five mixed peptide samples for ICAs, and four mixed peptide samples for ITAs. Prior to proteomic analysis, ICA and ATA peptide mixtures were each fractionated into five fractions by high pH reversed phase chromatography virtually as previously described [22], whereas the ITA peptide mixtures were fractionated into two fractions by hydrophilic interaction liquid chromatography (HILIC) fractionation essentially as described by Beck et al*.* [23].

### Proteomic analysis of tandem mass -tagged peptide fractions

The mass spectrometric analyses of ITA fractions were performed on a Thermo Scientific Orbitrap Q-Exactive (Thermo Fisher Scientific, Waltham, Massachusetts, USA) instrument coupled to a DionexUltiMate 3000 nano high-performance liquid chromatography (Thermo Fisher Scientific, Waltham, Massachusetts, USA), essentially as described previously [23], whereas the proteomic analysis of ATA and ICA fractions were performed on a Thermo Scientific Eclipse Orbitrap tribride mass spectrometer (Thermo Fisher Scientific, Waltham, Massachusetts, USA), coupled to a DionexUltiMate 3000 nano high-performance liquid chromatography, also as previously described [24].

### Data analysis

The data files from ICA and ATA experiments were analysed in Proteome Discoverer 2.4.0.305 (Thermo Fisher Scientific, Waltham, Massachusetts, USA). The MSPepSearch and Sequest HT processing nodes, both integrated with Proteome Discoverer, were used to search the data with the following criteria-protein database: UniProt/SwissProt database (downloaded 30/09/2019, containing 42369 sequences) and restricted to humans.

A human TMT spectral library of 401,168 unique peptides of high quality from millions of peptide-spectrum matches in tens of profiling projects, matching to 14,048 nonredundant proteins (13,953 genes) [25] was used with MSPepSearch. Fixed search parameters included trypsin, one missed cleavage allowed, TMT-six plex modification and carbamidomethylation at cysteine, while methionine oxidation, N-terminal carbamylation and N-terminal acetylation were set as dynamic. Precursor mass tolerance was 15 ppm for MSPepSearch searches and 8 ppm for Sequest HT searches. MSMS tolerance was set to 15 ppm for MSPepSearch and 0.05 Da for Sequest HT. False discovery rate (*fdr*) was calculated using a decoy database search and only high confidence peptide identifications (*fdr* < 1%) were included. The ITA data files were analysed as previously described [23].

Fisher’s Exact test to assess whether the pre-defined BM protein set was regulated was performed using the number of upregulated BM proteins (fold-change > 1, *p* ≤ 0.05), the number of unregulated BM proteins (fold-change > 1, *p* > 0.05), the number of upregulated non-BM proteins (fold-change > 1, *p* ≤ 0.05), and the number of unregulated non-BM proteins (fold-change > 1, *p* > 0.05).

Explorative proteomics data were analysed by Student’s t-test for each protein and subsequent *fdr* correction for multiple testing. Gene ontology (GO) enrichment analysis [26, 27] was performed using default settings of the DAVID Bioinformatics Resources [28, 29]. For this analysis, we defined proteins with a *p* ≤ 0.1 as regulated.

## Results

### Patient characteristics

Clinical characteristics and prescribed medication of the 55 patients included in the study are shown in Table 1. The investigated groups were all dominated by male sex. The T2DM groups had on average higher HbA1c, but overall, they did not differ from non-DM controls regarding other biochemical measures or blood pressure. Metformin was the most used anti-diabetic drug in all T2DM groups. There was no difference between T2DM and non-DM patients in the use of statins, but the T2DM ICA donors had a slightly higher use of antihypertensives than non-DM patients.

**Table 1:** Patient characteristics

Clinical characteristics	Internal thoracic artery (ITA)			Internal carotid artery (ICA)			Ascending thoracic aorta (ATA)		
	Non-DM	T2DM	*p*	Non-DM	T2DM	*p*	Non-DM	T2DM	*p*
*n*	7	9	NA	9	10	NA	10	10	NA
Age at surgery (y)	65 ± 12	68 ± 12	0.65	69 ± 5	71 ± 9	0.59	71 ± 8	64 ± 11	0.11
Male sex (%)	7 (100%)	8 (89%)	0.38	8 (80%)	8 (89%)	0.60	10 (100%)	10 (100%)	–
SBP (mmHg)	131 ± 9	139 ± 10	0.20	152 ± 22	148 ± 20	0.71	155 ± 16	142 ± 14	0.09
DBP (mmHg)	73 ± 8	74 ± 6	0.84	77 ± 10	74 ± 10	0.49	79 ± 7	75 ± 8	0.35
BMI (kg/m^2^)	30 ± 4	37 ± 4	0.01	27 ± 4	28 ± 3	0.35	28 ± 3	31 ± 6	0.18
Comorbidities									
Hypertension, *n* (%)	6 (86%)	7 (78%)	0.69	5 (50%)	8 (89%)	0.08	7 (70%)	10 (100%)	0.07
Stroke, *n* (%)	0 (0%)	0 (0%)	-	5 (50%)	3 (33%)	0.47	0 (0%)	0 (0%)	–
ACS, *n* (%)	0 (0%)	1 (11%)	0.38	1 (10%)	1 (11%)	0.95	0 (0%)	0 (0%)	–
Cancer, *n* (%)	1 (14%)	1 (11%)	0.86	1 (10%)	1 (11%)	0.95	0 (0%)	0 (0%)	–
Uraemia, *n* (%)	0 (0%)	0 (0%)	–	0 (0%)	0 (0%)	–	0 (0%)	0 (0%)	–
Never smoked, *n* (%)	2 (29%)	1 (11%)	0.38	3 (30%)	3 (33%)	0.89	4 (40%)	2 (20%)	0.34
Biochemical factors									
HbA1c (mmol/mol)	39.8 ± 4.3	61.7 ± 10.7	< 0.01	37.7 ± 2.8	54 ± 0.8	< 0.01	37.1 ± 2.8	63.1 ± 15.6	< 0.01
Cholesterol (mmol/L)	4.1 ± 0.6	3.6 ± 1.0	0.31	4.3 ± 1.2	3.9 ± 0.3	0.41	4.2 ± 1.3	3.9 ± 1.0	0.60
LDL (mmol/L)	2.2 ± 0.6	1.9 ± 0.8	0.45	2.7 ± 1.0	2.1 ± 0.3	0.18	2.5 ± 1.3	1.8 ± 0.3	0.22
HDL (mmol/L)	1.1 ± 0.3	0.9 ± 0.3	0.28	1.2 ± 0.3	1.1 ± 0.3	0.76	1.2 ± 0.3	1.1 ± 0.6	0.86
Triglyceride (mmol/L)	1.8 ± 1.1	1.8 ± 0.4	0.96	2.1 ± 0.9	1.9 ± 0.8	0.76	1.3 ± 0.2	2.0 ± 0.7	0.06
Creatinine (mmol/L)	93 ± 19	94 ± 22	0.91	80 ± 17	93 ± 30	0.42	88 ± 14	93 ± 17	0.55
Medication									
Metformin, *n* (%)	0 (%)	7 (78%)	< 0.01	0 (%)	7 (78%)	< 0.01	0 (%)	9 (90%)	< 0.01
Insulin, *n* (%)	0 (%)	3 (33%)	0.10	0 (%)	6 (66%)	< 0.01	0 (%)	0 (%)	–
Statins, *n* (%)	6 (86%)	7 (78%)	0.69	6 (60%)	8 (89%)	0.16	9 (90%)	9 (90%)	1.0
Antihypertensives, *n* (%)	7 (100%)	9 (100%)	–	6 (60%)	9 (100%)	0.04	8 (80%)	10 (100%)	0.15
Anticoagulants, *n* (%)	7 (100%)	8 (89%)	0.38	10 (100%)	9 (100%)	–	1 (10%)	10 (100%)	< 0.01
Steroids, *n* (%)	0 (%)	0 (%)	–	0 (%)	0 (%)	–	0 (%)	0 (%)	–

Results are depicted as number (n), percentage (%) or mean ± standard deviation. Student’s t-test was used for continuous variables. Pearson Chi-Square Test was used for dichotomous variables

Non-DM: non-diabetes mellitus; T2DM: type 2 diabetes mellitus; SBP: systolic blood pressure; DBP: Diastolic blood pressure; BMI: Body mass index; ACS: Acute coronary syndrome; HbA1c: Hemoglobin A1c; LDL: Low-density lipoprotein; HDL: High-density lipoprotein; NA: not available

### The medial layers of non-atherosclerotic arteries from T2DM patients are enriched in BM proteins

To test the hypothesis that BM accumulation occurs in large elastic arteries in T2DM patients, we analysed bulk ATA FFPE sections by LC–MS/MS. These specimens consist of > 90% media, and sparse intima and adventitia (as assessed by histology). We found 7 (LAMB2, LAMA5, COL4A2, COL18A1, HSPG2, NID1 and NID2) of 12 pre-defined BM proteins (COL4A1, COL4A2, COL18A1, LAMA2, LAMA4, LAMA5, LAMB1, LAMB2, LAMC1, NID1, NID2, HSPG2) to be significantly upregulated (*p* ≤ 0.05) in ATAs from T2DM patients (Fig. 1B). Fisher’s Exact test showed significant upregulation of BM proteins as a whole (*p* < 0.0001).

The medial- and adventitial layers of ITAs were analysed separately by LCM followed by LC–MS/MS. In T2DM patients, the medial layer showed a trend toward enrichment in BM proteins, although only COL4A2 was significantly upregulated (*p* ≤ 0.05) (Fig. 1C). In the adventitial layer of ITAs, BM proteins were unaffected (Fig. 1D). Although numerous components of interstitial extracellular matrix (e.g*.* collagens and glycoproteins) were detected in the analysis of ATAs and ITAs, only BM proteins appeared to be affected in arteries of T2DM patients.

In atherosclerotic plaques and the underlying medial layer of ICAs, however, we did not observe marked effects on BM proteins in T2DM patients, which may reflect the high level of variation as compared to ITAs and ATAs. COL18A1 (BM protein) was significantly upregulated in the atherosclerotic plaque (Fig. 1E), while NID2 (BM protein) was significantly downregulated in the underlying medial layer (Fig. 1F).

### Exploratory proteomic analysis reveals proteins regulated in arteries of T2DM patients

We next analysed the proteome data by explorative approaches. First, we ranked proteins from each specimen type based on *p*-values. The top five ranked proteins for each specimen type are shown in Table 2 and displayed on volcano plots in Fig. 1. In the plaque-associated medial layer of ICAs, Isoform 2 of heterogeneous nuclear ribonucleoprotein K (HNRNPK) was significantly upregulated post *fdr*-correction for multiple testing (*fdr* = 5.6·10^–4^), while 60S ribosomal protein L23 (RPL23) was downregulated at a near to significant level (*fdr* = 0.07) (Fig. 1F).

**Table 2:** Top five significantly regulated proteins in artery tissues/compartments from patients with type 2 diabetes mellitus

Protein name	Accession	Fold change	*p*	*fdr*
Ascending thoracic aorta (ATA)				
Unconventional myosin-Id (MYO1D)	O94832	0.88	5.0E−04	0.83
Collagen alpha-1(VIII) chain (COL8A1)	P27658	0.80	1.0E−03	1.00
NADH-cytochrome b5 reductase 3 (CYB5R3)	P00387	0.89	2.1E−03	1.00
Prenylcysteine oxidase 1 OS = Homo sapiens (PCYOX1)	Q9UHG3	0.91	2.5E−03	1.00
Trypsin-3 (PRSS3)	P35030	1.20	2.8E−03	1.00
Internal thoracic artery (ITA)—Medial layer				
Unconventional myosin-Ic (MYO1C)	O00159	1.18	1.3E−02	1.00
Desmin (DES)	P17661	1.28	2.0E−02	1.00
Histone H2A (H2AFX)	P16104	1.29	2.4E−02	1.00
Annexin A1(ANXA1)	P04083	0.85	2.5E−02	1.00
I soform 2 of Tropomyosin beta chain (TPM2)	P07951-2	1.27	2.7E−02	1.00
Internal thoracic artery (ITA)—Adventitial layer				
Prelamin-A/C (LMNA)	P02545	0.84	7.9E−03	1.00
Transforming growth factor beta-1-induced transcript 1 protein (TGFB1I1)	O43294	0.73	1.6E−02	1.00
Ig alpha-1 chain C region (IGHA1)	P01876	1.42	2.7E−02	1.00
Microtubule-associated protein 1B (MAP1B)	P46821	0.64	3.0E−02	1.00
Myosin-10 (MYH10)	F8VTL3	0.78	3.1E−02	1.00
Internal carotid artery (ICA)—Plaque				
Plastin-3 (PLS3)	P13797	0.50	5.3E−04	0.83
Immunoglobulin J chain (JCHAIN)	P01591	1.98	1.0E−03	1.00
Guaninenucleotide-binding protein G(i) subunit alpha-2 (GNAI2)	P04899	0.75	4.4E−03	1.00
Insulin-like growth factor II (IGF2)	P01344	1.40	5.1E−03	1.00
Alpha-parvin (PARVA)	Q9NVD7	1.81	5.2E−03	1.00
Internal carotid artery (ICA)—Media				
Isoform 2 of Heterogeneous nuclear ribonucleoprotein K (HNRNPK)	P61978-2	1.44	3.4E−07	5.6E−04
60S ribosomal protein L23 (RPL23)	P62829	0.29	4.4E−05	0.07
Ribonuclease 4 (RNASE4)	P34096	1.57	9.7E−04	1.00
Procollagen-lysine,2-oxoglutarate 5-dioxygenase 1 (PLOD1)	Q02809	0.50	2.0E−03	1.00
Calponin-2 (CNN2)	Q99439	2.04	2.0E−03	1.00

The table shows the top five most significantly regulated proteins for each tissue type/compartment in the study. Foldchanges are protein abundance in tissue from type 2 diabetes mellitus (T2DM) patients relative to tissue from non-diabetes mellitus (non-DM) patients

We next performed an unassisted GO enrichment analysis as an alternative explorative approach. GO enrichment analysis is a bioinformatic tool used to obtain an unbiased probability about alterations in constellations of proteins that may represent signalling pathways, clusters of interacting proteins or similar topographically situated proteins within an omics-dataset [26, 27]. Although no GO terms passed correction for multiple testing, several GO terms were nominally regulated for all investigated tissue types. Indeed, we identified “basement membrane” to be significantly upregulated (*p* = 0.0342) in ATAs (Table 3).

**Table 3:** Gene ontology enrichment analysis

Artery	Compartment	Regulation in T2DM	GO term	*p*	Proteins
ATA	–	Up	Basement membrane	0.034	LAMB2, LAMA5, COL5A1, COL18A1, HSPG2, NID2, LAMC1, NID1
		Down	Translation	0.007	RPL18, RPS23, GCN1, SLC25A11, RPL18A, SLC25A3, RPS8, RPL32, RPS11, RPS24, RPL21
ITA	Adventitia	Up	Blood microparticle	0.006	ALB, TF, IGHA1, IGHG1
ICA	Media	Up	rRNA binding	0.031	RPL37, RPL8, RPL11, RPS18
			Immune response	0.034	IGHV3, C1R, CXCL12, IGKV2-40, THBS1, C8B, C3
	Plaque	Up	Membrane attack complex	0.045	C6, C7, C8A, C9
		Down	Blood coagulation, fibrin clot formation	0.007	FGA, FGB, FGG
			Proteinaceous extracellular matrix	0.038	COL15A1, FBN1, FBLN2, EMILIN2, LUM, WISP2

No gene ontology (GO) terms were enriched in the medial layer of internal thoracic arteries (ITAs), and in the media underlying atherosclerotic plaques in internal carotid arteries (ICAs) among downregulated proteins

ATA: ascending thoracic aorta; ITA: internal thoracic artery; ICA: internal carotid artery; T2DM: Type 2 diabetes mellitus

In the atherosclerotic plaque and underlying media of ICAs, GO terms “Membrane attack complex” and “Immune response” displayed nominal upregulation, respectively, in T2DM patients (Table 3). Both GO terms include components of the complement system, although no individual complement factor showed overlap between these two compartments (Fig. 1E, F). We also found the GO term “Blood coagulation, fibrin clot formation” including the ⍺-, β- and ɣ-chain (FGA, FGB, FGG) of fibrinogen to be nominally downregulated in plaques of T2DM patients (Fig. 1E).

## Discussion

To the best of our knowledge, this is the first study to investigate the effect of T2DM on the artery proteome in several artery types in parallel. The primary focus of the study was to assess effects on BM proteins, as we previously found BM to be enriched in bulk ITA samples from T2DM patients [15].

In the present study, we provide evidence that BM accumulation in T2DM patients is not confined to ITAs (a medium-sized muscular artery), but also occurs in large elastic arteries exemplified by the ATA. This supports our hypothesis that BM accumulation is a general phenomenon of both micro- and macro-vessels in T2DM. Moreover, the concept is compatible with a recent report showing upregulation of collagen type IV and increased thickness of BM in small arteries in human vessel organoids in a model of diabetic vasculopathy [30]. The mechanism(s) leading to increased level of BM in small vessels in diabetic patients remain elusive. In vitro, treating endothelial cells or VSMCs with high glucose or serum from diabetic patients increases expression of BM proteins [31–36]. Moreover, non-enzymatical and oxidative BM protein modifications associated with diabetes [37, 38] are believed to affect BM turnover rates, leading to accumulation over time [39]. The relative contribution of these possibilities will have to await further studies.

The major components of the BM are collagen type IV, laminins, nidogens, and heparan-sulfate proteoglycans, primarily Heparan-sulfate proteoglycan 2 (HSPG2) and COL18 [40, 41]. We pre-defined a list of vascular BM proteins including COL4A1 and COL4A2 (the ⍺-chains of the dominant vascular COL4 subtype), all laminins detectable by MS in arteries (LAMA2, LAMA4, LAMA5, LAMB1, LAMB2, and LAMC1), both nidogens (NID1 and NID2), HSPG2 and COL18A1 (we do not detect other COL18 ⍺-chains in artery tissue). Despite a limited number of samples, 7 of these 12 pre-defined BM proteins were significantly upregulated in ATAs, and unassisted GO enrichment analysis highlighted “basement membrane” as the only significantly enriched GO term in T2DM aortas among upregulated proteins emphasizing that BM changes in T2DM patient arteries are predominant as compared to other GO terms. The medial layer constitutes > 90% of the ATA cross-sectional area, and BM enrichment therefore most likely occurs in this compartment. This notion is supported by our separate proteomic profiling of the medial- and adventitial layers of ITAs, where we observed a trend toward BM enrichment in the medial layer (only COL4A2 is significant), but not in the adventitia. Moreover, the medial layer holds by far the majority of BM in arteries as compared to the intima and adventitia [15] (Additional file 2: Figure S2).

In contrast to the homogenous, non-lesioned arteries (ATAs and ITAs), we did not observe BM enrichment in ICA plaques or in the underlying media from T2DM patients. The main reason for this observation is likely a much higher variation in atherosclerosis samples. The age, cellular and matrix content, i.e. the phenotype of atherosclerotic plaques vary tremendously from individual to individual, Moreover, atherosclerotic plaques are extremely heterogenous within the same person and the choice of histological area selected for LCM has profound effects on the biochemical results. Indeed, issues with high variability for this tissue type is in line with a previous report based on atherosclerotic tissue in diabetes. Although Edsfeldt et al*.* included almost 200 atherosclerosis samples, large variation of all identified components (cell types, fibrosis, fatty streak, etc.) was observed making it impossible to determine if alterations were present in diabetes [42]. Even though we attempt to reduce variation by LCM based microdissection variation may therefore likely mask possible T2DM-related effects on the arterial proteome.

The pathophysiological significance of BM accumulation in T2DM is not clear, however a robust indication of a causal link between the level of artery BM and cardiovascular disease comes from the highly replicated GWAS-observation of associations between non-coding *COL4A1/A2* variants and coronary- and peripheral artery disease [16–18]. These associations appear to be relevant also in the diabetic population [43]. The explanation behind this observation is not known, but it is important to realize that diabetes-associated artery effects are not limited to the presence of increased atherosclerotic plaques per se, but include also general artery alterations: Increased artery stiffening [7, 44], medial calcification [5, 6], and impaired expansive remodelling in response to plaque growth [9] (an observation in line with the paradoxically reduced incidence of aneurysms in diabetes [4]). The underpinnings of these diabetes-induced effects have yet to be understood, but we hypothesize that BM-accumulation may be a contributing factor. The arterial BM is abundant underlining the endothelium and encapsulating VSMCs throughout the medial layer [15, 16], and in vitro studies have demonstrated the importance of the BM on vascular cell differentiation and function [45, 46]. In support of our hypothesis, we recently observed that genetic reduction of Col4a1/a2 in mice resulted in VSMC de-differentiation and accelerated development of abdominal aortic aneurysms [47], a condition which diabetic patients is partly protected against [48]. Importantly, this hypothesis requires further testing in future studies.

Although the present study is large compared to the limited number of previously published arterial proteome studies, the exploratory part of our study is limited by low sample numbers. Nevertheless, we identified 106 proteins in ATAs, 90 in ICAs and 18 in ITAs displaying nominal significant differences between T2DM and non-DM patients. HNRNPK was upregulated in the media underlying atherosclerotic plaques in ICAs, and was the only protein to surpass the *fdr*-corrected significance level. HNRNPK is a member of the pre-mRNA-binding protein family [49] and regulates gene expression at both transcriptional and post-transcriptional levels. Previously, it was observed that this protein is upregulated in serum-activated vascular smooth muscle cells [50] and that it accumulates in experimental atherosclerosis [51]. Moreover, it is partly responsible for the effects of insulin on the regulation of angiotensinogen in the vasculature [52]. Combined with the observations from the present study, this suggests that HNRNPK may be involved in atherosclerosis among patients with diabetes.

Also, in the medial layer underlying ICA plaques, RPL23 showed near *fdr*-corrected significant downregulation. RPL23 is involved in apoptosis [53], but putative roles in arterial diseases in diabetes remain to be investigated.

Besides our finding that the GO term “basement membrane” was significantly enriched among upregulated proteins in ATAs, we identified additional GO terms to be enriched in arteries of T2DM patients. “Membrane attack components”, i.e*.* complement factors, displayed nominal upregulation in plaques from T2DM patients. Although this finding requires validation, this information is new since no previous proteome investigations have been performed on plaques from T2DM patients, and no studies have previously investigated these proteins in atherosclerotic plaques in the context of diabetes. Interestingly, complement factors have been shown to be involved in the development of small vessel disease in diabetes [54, 55]. Reactions against non-enzymatic glycaemic proteins have been suggested as trigger elements of complement activation. The GO enrichment analysis also showed that the GO term “Blood coagulation, fibrin clot formation” was enriched among downregulated proteins in ICA plaques, and fibrinogens FGA, FGB and FGG were the three proteins showing the largest reduction in plaques from T2DM patients.

This study has several limitations. First, our investigations are based on tissue from individuals, who have undergone elective cardiovascular surgery and therefore suffer from a certain degree of atherosclerotic disease. Whether the results from this preselected group are applicable to healthy individuals is unknown. Second, the majority of included individuals were males. This may introduce a gender bias to the study. Third, previous studies have demonstrated that arteries from T2DM patients are less compliant possibly by increased cross-linking of extracellular matrix constituents [56, 57]. Cross-linking may affect protein extraction and trypsination efficiency, and we cannot exclude that this provides a bias in our results. Lastly, our dataset is biased toward identification of proteins that are abundant in artery tissues, e.g*.* structural extracellular matrix components. As such, there will be effects on the artery proteome in T2DM patients, which we cannot detect.

## Conclusion

The present study shows that BM proteins accumulate in large elastic arteries (i.e*.* ATAs, largely composed of media) as well as in the medial layer of muscular arteries (i.e*.* ITAs) from patients with T2DM. Our findings thereby extend the long-recognized accumulation of BM in smaller vessels in diabetic patients [58–60]. We hypothesize that this effect plays a role in development of arterial disease in T2DM patients.

In addition, our explorative results point to new groups of proteins that may be altered in arteries of T2DM patients, however, these findings required validation in future studies.

## Supplementary Information

**Additional file 1: Figure S1.** Representative Masson trichrome and Weigert stainings of internal thoracic arteries (ITA), ascending thoracic aorta (ATA) and internal carotid artery with atherosclerotic plaque (ICA). Intima, media, adventitial and plaque compartments are shown as indicated.

**Additional file 2: Figure S2.** Representative Collagen IV stainings of internal thoracic arteries (ITA), ascending thoracic aorta (ATA) and internal carotid artery with atherosclerotic plaque (ICA). Intima, media, adventitia and plaque compartments are shown as indicated.

## Data Availability

The datasets generated and/or analysed during the current study are not publicly available due to hospital guidelines and legislation regarding personal data. Data will be available from the corresponding author on reasonable request and with permission of Odense University Hospital Legal Department.

## Abbreviations

ATA: Ascending thoracic aorta
BM: Basement membrane
CVD: Cardiovascular disease
DM: Diabetes mellitus
DTT: Dithiotreitol
EDTA: Ethylenediamine tetraacetic acid
FDR: False discovery rate
FGA: Fibrinogen, alpha-chain
FGB: Fibrinogen, beta-chain
FGG: Fibrinogen, gamma-chain
GO: Gene ontology
GWAS: Genome-wide association study
HILIC: Hydrophilic interaction liquid chromatography
HNRNPK: Heterogeneous nuclear ribonucleoprotein K
ICA: Internal carotid artery
ITA: Internal thoracic artery
IAA: Iodoacetamide
LCM: Laser capture microdissection
LC–MS/MS: Liquid chromatography tandem mass spectrometry
OAB: Odense Artery Biobank
RPL23: 60S ribosomal protein L23
SDS: Sodium dodecyl sulphate
SNP: Single nucleotide polymorphism
T2DM: Type 2 diabetes mellitus
TEAB: Triethyl ammonium bicarbonate
