# Supplementary material for: Basement membrane proteins in various arterial beds from individuals with and without type 2 diabetes mellitus: a proteome study
Source: Cardiovasc Diabetol. 2021 Sep 8;20:182. doi: 10.1186/s12933-021-01375-7 (PMC8428091; doi:10.1186/s12933-021-01375-7)
Supplement: Supplementary file 1 — Additional file 1: Figure S1. Representative Masson trichrome and Weigert stainings of internal thoracic arteries (ITA), ascending thoracic aorta (ATA) and internal carotid artery with atherosclerotic plaque (ICA). Intima, media, adventitial and plaque compartments are shown as indicated. [file 12933_2021_1375_MOESM1_ESM.pdf]

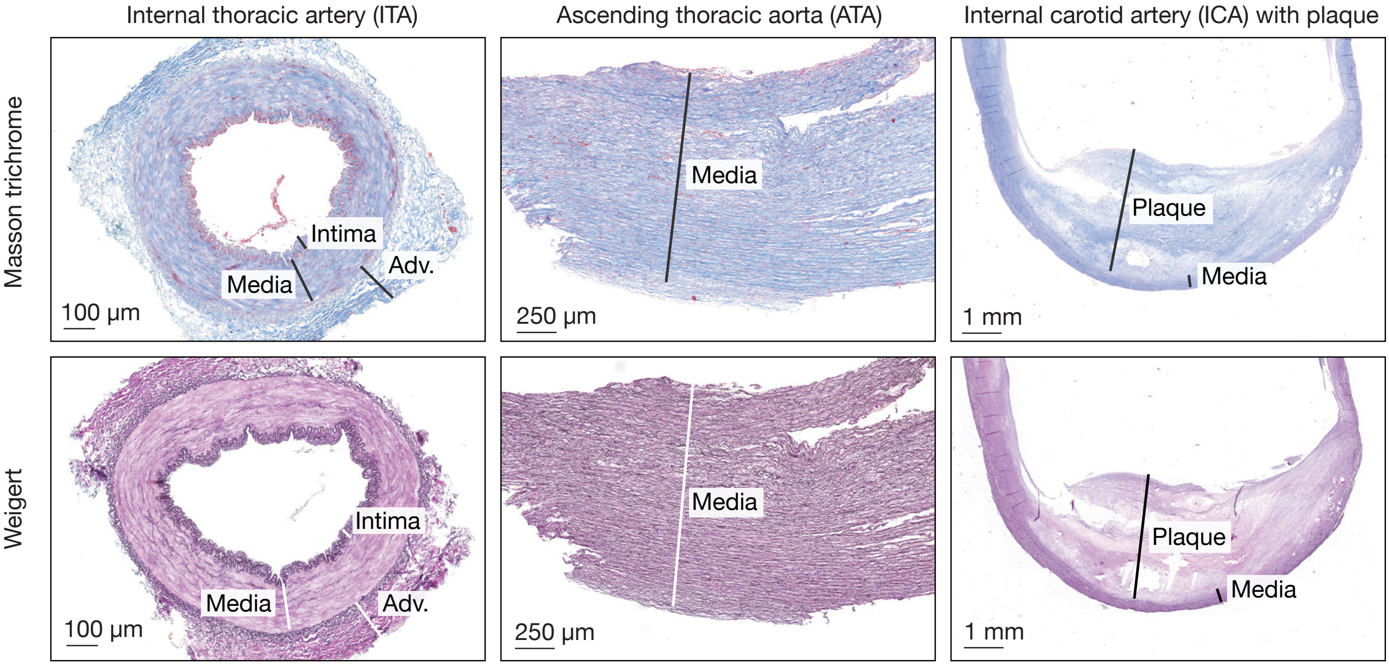
Internal thoracic artery (ITA)
Ascending thoracic aorta (ATA)
Internal carotid artery (ICA) with plaque
Masson trichrome
Weigert
Intima
Media
Adv.
100 µm
Media
250 µm
Plaque
Media
1 mm
Intima
Media
Adv.
100 µm
Media
250 µm
Plaque
Media
1 mm
